# Supplementary material for: Splice-Junction-Based Mapping of Alternative Isoforms in the Human Proteome
Source: Cell Rep. Author manuscript; Available in PMC 2020 Jan 15. (PMC6961840; doi:10.1016/j.celrep.2019.11.026)
Supplement: 2 [file NIHMS1546469-supplement-2.zip › DF1/PXD010154/Prostate/Prostate_9_ERMP1_FLQEILLISDSYPK.pdf]

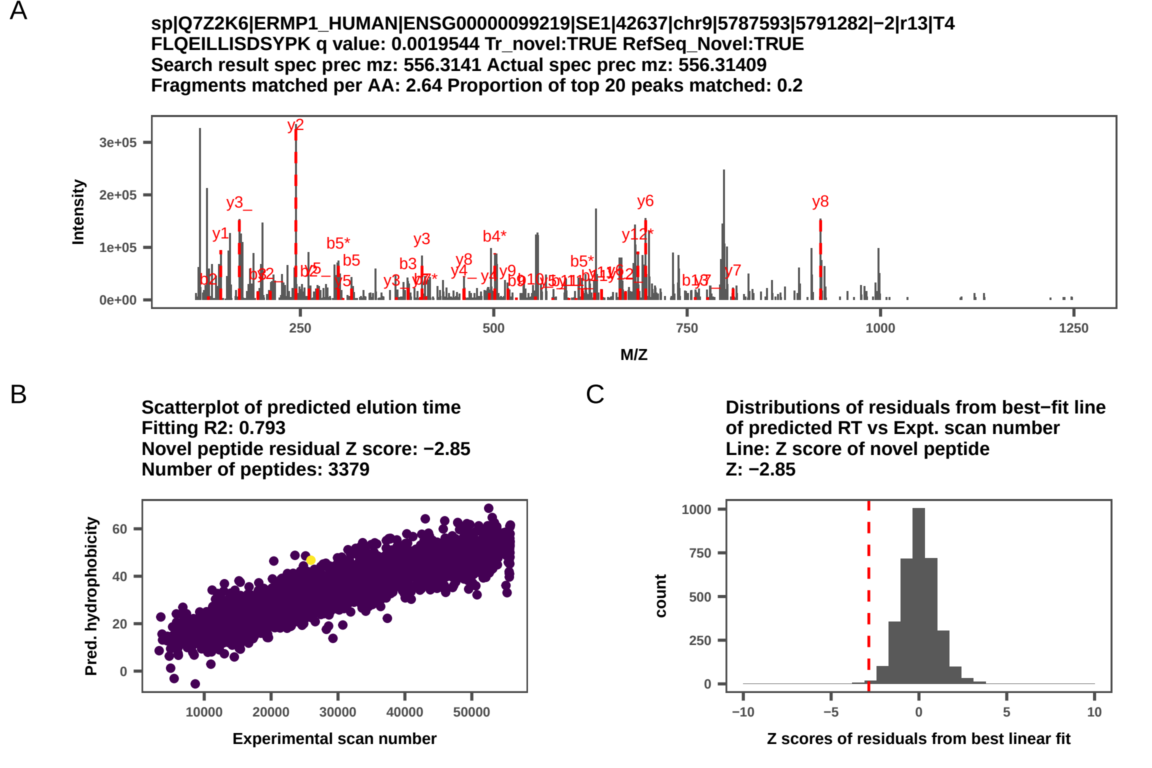

A
sp|Q7Z2K6|ERMP1_HUMAN|ENSG00000099219|SE1|42637|chr9|5787593|5791282|−2|r13|T4
FLQEILLISDSYPK q value: 0.0019544 Tr_novel:TRUE RefSeq_Novel:TRUE
Search result spec prec mz: 556.3141 Actual spec prec mz: 556.31409
Fragments matched per AA: 2.64 Proportion of top 20 peaks matched: 0.2
Intensity
3e+05
2e+05
1e+05
0e+00
y2
y3_
y1
b2
b5*
b5
y5
y3
b3
b4*
y8
y4
y9
y6
y12*
y8
b5*
y7
M/Z
250
500
750
1000
1250
B
Scatterplot of predicted elution time
Fitting R2: 0.793
Novel peptide residual Z score: −2.85
Number of peptides: 3379
Pred. hydrophobicity
60
40
20
0
10000
20000
30000
40000
50000
Experimental scan number
C
Distributions of residuals from best−fit line
of predicted RT vs Expt. scan number
Line: Z score of novel peptide
Z: −2.85
count
1000
750
500
250
0
−10
−5
0
5
10
Z scores of residuals from best linear fit
